# Engineering TGF-β inhibitor-encapsulated macrophage-inspired multi-functional nanoparticles for combination cancer immunotherapy

**DOI:** 10.1186/s40824-023-00470-y

**Published:** 2023-12-18

**Authors:** Jaehyun Kim, Minjeong Kim, Seok-Beom Yong, Heesoo Han, Seyoung Kang, Shayan Fakhraei Lahiji, Sangjin Kim, Juhyeong Hong, Yuha Seo, Yong-Hee Kim

**Affiliations:** 1 Department of Bioengineering, Institute for Bioengineering and Biopharmaceutical Research, Hanyang University, Seoul, 04763 Republic of Korea; 2 Nucleic Acid Therapeutics Research Center, Korea Research Institute of Bioscience and Biotechnology (KRIBB), Chungcheongbuk-do, 28116 Republic of Korea; 3 Institute for Bioengineering and Biopharmaceutical Research (IBBR), Hanyang University, Seoul, 04763 Republic of Korea; 4 Cursus Bio Inc. Icure Tower, Gangnam-gu, Seoul, 06170 Republic of Korea

**Keywords:** Cancer immunotherapy, Tumor-associated macrophage, Immune cell-inspired nanoparticle, TGF-β inhibition, Immune checkpoint inhibitor, Combination therapy

## Abstract

**Background:**

The emergence of cancer immunotherapies, notably immune checkpoint inhibitors, has revolutionized anti-cancer treatments. These treatments, however, have been reported to be effective in a limited range of cancers and cause immune-related adverse effects. Thus, for a broader applicability and enhanced responsiveness to solid tumor immunotherapy, immunomodulation of the tumor microenvironment is crucial. Transforming growth factor-β (TGF-β) has been implicated in reducing immunotherapy responsiveness by promoting M2-type differentiation of macrophages and facilitating cancer cell metastasis.

**Methods:**

In this study, we developed macrophage membrane-coated nanoparticles loaded with a TGF-βR1 kinase inhibitor, SD-208 (M$\phi$-SDNP). Inhibitions of M2 macrophage polarization and epithelial-to-mesenchymal transition (EMT) of cancer cells were comprehensively evaluated through in vitro and in vivo experiments. Bio-distribution study and in vivo therapeutic effects of M$\phi$-SDNP were investigated in orthotopic breast cancer model and intraveneously injected metastasis model.

**Results:**

M$\phi$-SDNPs effectively inhibited cancer metastasis and converted the immunosuppressive tumor microenvironment (cold tumor) into an immunostimulatory tumor microenvironment (hot tumor), through specific tumor targeting and blockade of M2-type macrophage differentiation. Administration of M$\phi$-SDNPs considerably augmented the population of cytotoxic T lymphocytes (CTLs) in the tumor tissue, thereby significantly enhancing responsiveness to immune checkpoint inhibitors, which demonstrates a robust anti-cancer effect in conjunction with anti-PD-1 antibodies.

**Conclusion:**

Collectively, responsiveness to immune checkpoint inhibitors was considerably enhanced and a robust anti-cancer effect was demonstrated with the combination treatment of M$\phi$-SDNPs and anti-PD-1 antibody. This suggests a promising direction for future therapeutic strategies, utilizing bio-inspired nanotechnology to improve the efficacy of cancer immunotherapy.

**Graphical Abstract:**

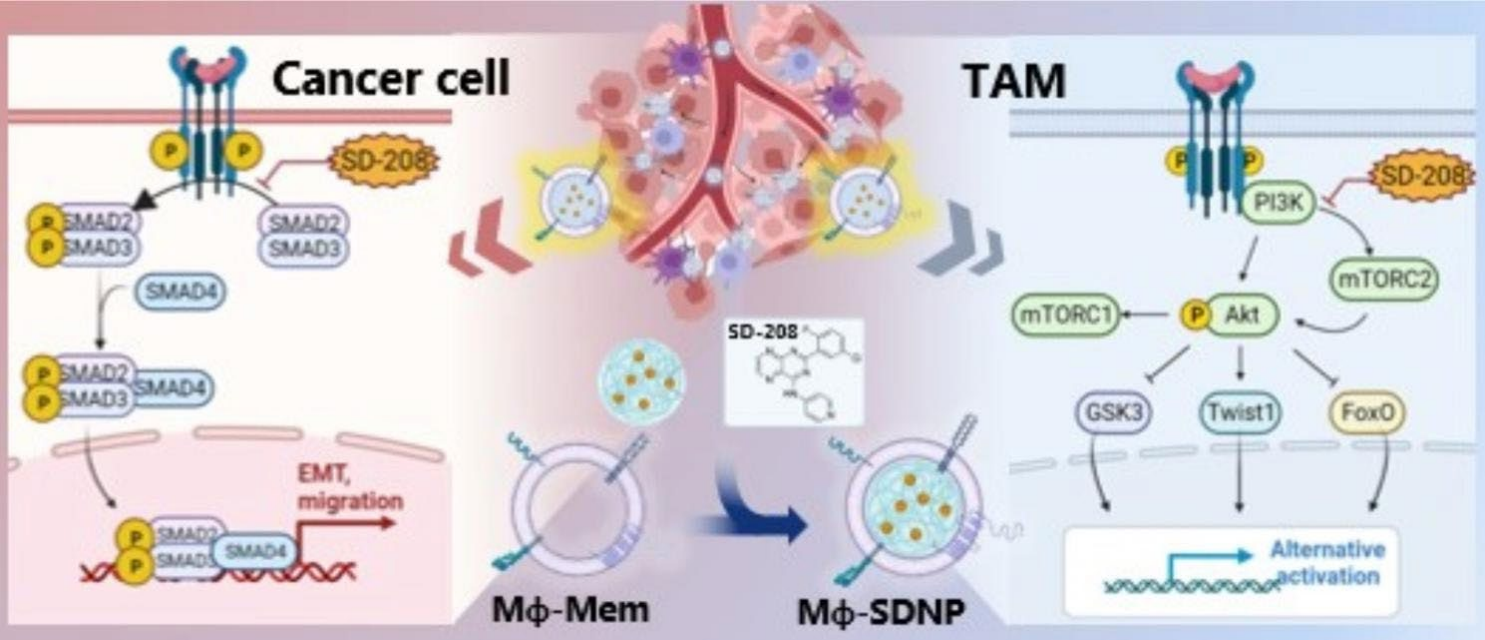

**Supplementary Information:**

The online version contains supplementary material available at 10.1186/s40824-023-00470-y.

## Background

In recent years, the advent of nanotechnology has broadened its scope in the field of targeted drug delivery, offering the advantages of enhanced tissue specificity and intracellular precision [1]. Furthermore, nanoparticle delivery has demonstrated efficacy in augmenting the physicochemical attributes of encapsulated agents and surmounting biological obstacles, thereby boosting safety and therapeutic effectiveness [2]. Nevertheless, the utility of nanoparticle delivery faces certain limitations for achieving clinical success, particularly the hindrances posed by immune clearance and off-target effects in the bloodstream, thus indicating a need for further improvement [3]. Cell membrane-coated nanoparticles, also referred to as cell-mimicking nanoparticles, offer synergistic benefits by integrating the physicochemical properties of traditional core nanoparticles with the biological features of cell membranes. This duality presents opportunities for enhancing anti-cancer efficacy while minimizing side effects [4]. Cell-mimicking nanotechnologies have been explored in diverse oncological domains, encompassing drug delivery, phototherapy, and immunotherapy [5–7]. The selection of the cell membrane typically depends on the intended target site and treatment strategy employed in nanoparticle delivery [8–10]. For instance, nanoparticles sheathed in red blood cell membranes can evade immune clearance in the blood, thereby enhancing bioavailability [11]. Moreover, nanoparticles enveloped with cancer cell membranes can be employed as cancer vaccines to deliver cancer antigens.

Emerging cancer immunotherapies, including immune checkpoint inhibitors, CAR-T cells, and therapeutic cancer vaccines, have shown promise as robust anti-cancer treatments. Of these, immune checkpoint inhibitors, which gained clinical success in the early 2010s, have significantly elevated patient survival rates, thereby sparking widespread interest [12]. These inhibitors offer an advantage over traditional chemotherapies by bolstering the host immune system defense against cancer and have thus become a standard-of-care in treatment. However, these therapies have shown effectiveness in only a subset of cancers, with lower than desired response rates and reports of immune-related adverse effects in certain patients [12]. As a result, a myriad of nanomedicines with immunotherapeutic potential has been explored recently to enhance efficacy and response rates, either as monotherapies or in combination therapies [13]. Despite significant strides in translational research, the subsequent clinical trials and approvals for next-generation immune checkpoint inhibitors have been scarce [14]. This has led to a renewed focus on identifying responsive patient populations for cancer immunotherapies, highlighting the need for innovative strategies to push the field forward [15]. Overcoming tumor-induced immunosuppression has emerged as a critical area of study, with extensive research being conducted on various immunosuppressive mechanisms within tumors [16].

The effectiveness of cancer immunotherapeutics and the ability of cancer cells to develop resistance are intimately associated with the constituents and interactions within the tumor microenvironment (TME) [17]. Tumor-associated macrophages (TAMs), in particular, are known to be key contributors to the immunosuppressive milieu of the tumor microenvironment [18]. Previous studies have indicated that cancer cells and cancer-associated fibroblasts co-opt monocytes and macrophages via the CCL2-CCR2 and CSF1-CSF1R signaling axis [19]. These recruited cells are then differentiated into M2-type macrophages, which primarily contribute to pro-tumorigenic tissue remodeling rather than phagocytosis of cancer cells. Moreover, the expression of integrin α4 and β1 on TAMs facilitates their interaction with VCAM-1 on cancer cells, thereby aiding the migration of cancer cells towards distant metastatic sites [20]. Also, the interaction of SIRPα on macrophages and CD47 on cancer cells inhibits the clearance of cancer cells via phagocytosis [19]. Given the ability of macrophages to infiltrate the tumor microenvironment and interact with various components therein, thus impacting the immune evasion and proliferation of cancer cells through multiple protein functions, we posited that targeting macrophages within the tumor microenvironment could suppress the immune evasion and proliferation of cancer cells. Importantly, the inhibition of the CCL2-CCR2 and CSF1-CSF1R axis is currently being investigated in clinical trials using various inhibitor types, including anti-CSF1R monoclonal antibodies, anti-CCL2 monoclonal antibodies, and small molecules [21].

Herein, we propose a cell-mimicking nanoplatform that employs a cell membrane coating strategy inspired by macrophage membrane protein function. We propose macrophage membrane-coated nanoparticles (MϕNP) can subvert the immunosuppressive activity of TAMs by scavenging immunosuppressive cytokines such as CCL2 and CSF1. Moreover, our findings suggest MϕNP can potentially restore the phagocytic activity of standard macrophages by obstructing the CD47-SIRPα interaction within the TAM in the tumor microenvironment. The homotypic targeting ability of MϕNP could enable the specific targeting of TAMs and metastasis-associated macrophages (MAMs) through integrins on macrophage membranes.

Transforming growth factor-beta (TGF-β) is a quintessential immunosuppressive factor within tumor regions. Recent studies have elucidated that TGF-β impedes T cell infiltration into tumor tissues and weakens the anti-tumor function of T cells, thereby diminishing the response rate to immune checkpoint inhibitors. Moreover, TGF-β signaling promotes the differentiation of macrophages into the M2-type, stiffens stromal cells, and induces epithelial-to-mesenchymal transition (EMT) in cancer cells, consequently enhancing their invasiveness to secondary sites [22, 23]. Accordingly, we designed nanoparticles loaded with SD-208, a TGF-βR1 kinase inhibitor, and coated them with a macrophage membrane (Mϕ-SDNP) to selectively target TAMs, MAMs, and cancer cells.

Through this approach, we aim to mitigate the M2-type differentiation of macrophages within tumor tissue, disrupt the interaction between cancer cells and macrophages, and suppress the invasiveness of cancer cells, thereby potentially curbing metastasis to secondary tumor sites and augmenting the effectiveness of cancer immunotherapies.

## Materials and methods

### Cell culture

Raw264.7 cells, a mouse macrophage cell line, and 4T1 cells, a murine breast cancer cell line originated in the Balb/c mouse strain, were purchased from American Type Culture Collection (ATCC, USA). Raw264.7 cells were cultured in high glucose Dulbecco’s Modified Eagle’s Medium(DMEM) and 4T1 cells were cultured in RPMI 1640 supplemented with both 10% fetal bovine serum (Welgene, Korea) and 1%(v/v) penicillin/streptomycin (Welgene, Korea).

Bone marrow-derived macrophages (BMDMs) were extracted by referring to experimental methods in previous studies. In brief, bone marrow cells were obtained from the femur and tibia of 6 to 8-week-old Balb/c mice (Orient bio, Korea) by flushing inside the bones with PBS. And bone marrow cells were treated with a macrophage differentiation medium, which was high glucose DMEM supplemented with 10% FBS and 1% penicillin/streptomycin, and 10ng/ml of M-CSF1 (R&D systems, USA). And 4 days after, half amount of macrophage differentiation medium was added. In order to differentiate to M2 type (M2-BMDM), 10ng/ml of IL-4 was added with macrophage differentiation medium.

### Macrophage membrane isolation and characterization

The macrophage membrane (Mϕ-mem) was isolated from Raw264.7 cells according to the previous method. In short, The grown raw 264.7cells were harvested and washed with phosphate-buffered saline (PBS). Then the cell pellet was suspended in a hypotonic solution containing 20mM Tris-HCl, 10mM KCl, 2mM MgCl_2,_ and an EDTA-free SIGMAFAST™ Protease Inhibitor cocktail (Sigma Aldrich, USA) and disrupted by sonication for 1 min at 10% amplitude, purse on 2s, purse off 5s. Then the solution was centrifuged with 20,000 g for 45 min. After that, the supernatant was centrifuged at 30,000 g for 45 min. Lastly, the supernatant was centrifuged at 110,000 g for 45 min, and pellets were obtained. The cell membrane was diluted and stored in water containing 0.2 mM EDTA.

### Preparation of PLGA nanoparticles & cell membrane coating

PLGA nanoparticles were prepared with o/w single emulsion. 1mL of PLGA (resomer® RG 503 H, Sigma Aldrich, USA) dissolved in dichloromethane (6 mg/ml) was added dropwise into 6ml of 0.5% PVA solution. SD-208 (Sigma Aldrich, USA) was mixed with PLGA solution with a polymer-to-drug ratio of 10:1. For the fluorescence imaging experiment, Cy5.5 NHS or DiD (Sigma Aldrich, USA) was loaded into the PLGA solution at 5% (w/w). The mixture was then agitated with 200RPM until the dichloromethane was evaporated entirely. After the dichloromethane was evaporated, the solution was centrifuged and washed twice with distilled water at 20,000 g for 20 min to remove the unloaded-free drugs or dyes Membrane-coated nanoparticles were prepared with the sonication method. In short, the cell membrane was mixed with PLGA NP with a 1:1 weight ratio (w/w) and sonicated in a water bath sonicator for 3 min. In order to remove the uncoated nanoparticles, the mixture was centrifuged at 20,000 g for 20 min.

### Characterization of membrane proteins on MϕNP

The presence or absence of the membrane proteins was evaluated via Western blot analysis. Macrophage lysate, isolated macrophage membranes, and MϕNP proteins were prepared, then the protein concentration of each sample was determined by the Bradford protein assay. Then 20ug of proteins were loaded on a 10% (w/v) SDS polyacrylamide gel. After electrophoresis, the obtained gel was transmitted to the PVDF membrane, and cell membrane proteins were evaluated.

Furthermore, MϕNP was deposited onto a carbon-taped grid and dried. After that, 1% uranyl acetate stain was added to the grid. After that, MϕNP was visualized using a transmission electron microscope. Energy-dispersive X-ray spectroscopy elemental mapping analysis was performed by Double Cs & Monochromated TEM installed at the Korea Basic Science Institute (KBSi), Seoul.

### Cytokine scavenging effect

The cytokine scavenging function of CCR2 and CSF1R on MϕNP was evaluated by ELISA. Anti-CCR2 antibodies (Abcam, UK) and Anti-CSF1R antibodies (Cell signaling technology, USA) were used to block CCR2 and CSF1R on MϕNPs (CCR2b- MϕNPs and CSF1Rb- MϕNPs). PLNPs, CCR2b(or CSF1Rb)- MϕNPs, and MϕNPs at final concentrations ranging from (0–800 μg/ml) were mixed with recombinant mouse CCL2 (Invitrogen, USA) or CSF1 proteins (R&D systems, USA). The mixtures were incubated at 37 °C for 2 h and centrifuged at 20,000 g, 4 °C for 30 min. Then CCL2(or CSF1)-bound MϕNPs, CCR2b (or CSF1Rb)- MϕNPs, and PLNPs went down to be pellet and unbound CCL2, and CSF1 proteins were in the supernatants. And these supernatants were analyzed by ELISA.

### Physicochemical characterization of Mϕ-SDNP

The prepared particles were diluted in distilled water and analyzed to measure the hydrodynamic size and surface charge via dynamic light scattering and zeta potential measurement systems (Zetasizer-Nano ZS, Malvern instrument, Worcestershire, UK) for comparing Mϕ-SDNP to PLNP, SDNP, MϕNP, and Mϕ membrane vesicle.

To measure encapsulation efficiency and drug loading of Mϕ-SDNP, SD-208 loaded Mϕ-SDNPs were dissolved in 1 mL of DMSO solution. The concentrations of SD-208 in the solution were detected by measuring absorbance at wavelengths of 370 nm (Infinite M200Pro, TECAN, Korea).

The encapsulation efficiency was calculated by the formular of

$$\begin{aligned} & \frac{{({\rm{weight}}\,{\rm{of}}\,{\rm{SD}} - 208\,{\rm{in}}\,{\rm{total}}\,{\rm{M}}\varphi - {\rm{SDNP}})\left( {{\rm{mg}}} \right)}}{{({\rm{total}}\,{\rm{weight}}\,{\rm{of}}\,{\rm{SD}} - 208\,{\rm{added}}\,{\rm{during}}\,{\rm{M}}\varphi - {\rm{SDNP}}\,{\rm{preparation}})\left( {{\rm{mg}}} \right)}}\\ &\quad {*}100\left( \% \right)\end{aligned}$$

And the drug loading was calculated by the formular of

$$\frac{{({\rm{weight}}\,{\rm{of}}\,{\rm{SD}} - 208\,{\rm{in}}\,{\rm{M}}\varphi - {\rm{SDNP}})\left( {{\rm{mg}}} \right)}}{{({\rm{weight}}\,{\rm{of}}\,{\rm{M}}\varphi - {\rm{SDNP}})\left( {{\rm{mg}}} \right)}}*100\left( \% \right)$$

To verify the release of SD-208, Mϕ-SDNP and SDNP were resuspended in PBS at pH 5.5 and pH 7.4, and they were incubated at 4℃ cold room with agitating. The particles were centrifuged at 20,000 g for 20 min, and the supernatants of samples were collected at 2, 4, 6, 12, 24, 48, and 72 h. The concentrations of SD-208 in each supernatant were detected by measuring absorbance at wavelengths of 370 nm (Infinite M200Pro, TECAN, Korea).

### In vitro cellular toxicity assay

MTT assay was performed to verify the extent of cell toxicity in vitro. Cells were seeded into the plate, and nanoparticles were treated for 18 h. Then MTT agent was added for 3 h until the purple precipitate was visible. Finally the relative absorbance was measured at 570 nm by treatment with DMSO (Infinite M200Pro, TECAN, Korea).

### In vitro anti-metastatic functional analysis of Mϕ-SDNP

The invasiveness of 4T1 cells on the plate was evaluated by wound-healing assay. For this assay, 90% confluenced 4T1 cells were scratched with SPLScar™ (SPL life science, Korea). And Each well was treated overnight with different groups (TGF-β :20ng/mL; particles: 10 μg/mL).

The extravasation ability of tumor cells (4T1, ATCC) was evaluated via trans-endothelial migration assay. For this assay, 1 × 10^5^ HUVEC cells in 100 μl Endothelial cell growth medium (LONZA, Switzerland) were seeded into the upper chamber of the transwell (8-μm pore filters) and cultured overnight at 37℃ to form an endothelial cell layer. The medium was eliminated, and then tumor cells (9 × 10^4^ cells in 200 μl medium) were plated into the upper chamber, followed by inoculation of medium containing M2-BMDM derived cytokines. Then tumor cells were treated with different sample (TGF-β: 20ng/mL; particles: 10 μg/mL) and incubated for 16 h at 37℃. Next, the upper chamber was washed with PBS twice, and migrated cells were fixed with 3.7% formaldehyde. Finally, the migrated cells were stained with crystal violet (1 mg/mL) for 15 min at room temperature and washed with PBS, followed by the removal of PBS with sterile cotton swabs. The migrated cells were observed with a microscope.

### In vitro EMT inhibition ability of Mϕ-SDNP

In order to analyze epithelial-to-mesenchymal transition (EMT) marker expression on tumor cells, qRT-PCR analysis and Western blot were conducted. 4T1 cells were seeded on the well plate and treated with different samples each other that contained SDNPs, MϕNPs, Mϕ-SDNPs (10 μg/mL) for 12 h at 37℃ to analyze EMT marker on transcription level via qRT-PCR. And cancer cells were treated with TGF-β (20ng/mL), except for the non-treat group, for 2 h at 37℃, and total RNA was isolated from cancer cells via RNeasy mini kits (Qiagen, Germany), and cDNA was synthesized by using iScript cDNA synthesis kits (Bio-Rad, USA). The mRNA expression was normalized by mouse GAPDH expression, and the EMT marker, such as zeb1, snail, twist, slug, mmp-2, and mmp-9 was calculated respectively by the ΔΔCt method.

**Table:** 

Target	Primer	Sequence (5’ – 3’)
gapdh	Forward	AATGGGCAGCCGTTAGGAAA
	Reverse	GCGCCCAATACGACCAAATC
zeb1	Forward	ACAAGACACCGCCGTCATTT
	Reverse	GCAGGTGAGCAACTGGGAAA
snail	Forward	CCACTGCAACCGTGCTTTT
	Reverse	CACATCCGAGTGGGTTTGG
twist	Forward	CGGGTCATGGCTAACGTG
	Reverse	CAGCTTGCCATCTTGGAGTC
slug	Forward	CATCCTTGGGGCGTGTAAGT
	Reverse	ATGGCATGGGGGTCTGAAAG
mmp2	Forward	GAGAACCAAAGTCTGAAGAG
	Reverse	GGAGTGAGAAGCTGATTAG
mmp9	Forward	TGCGACCACATCGAACTTCG
	Reverse	CCAGAGAAGAAGAAAACCCTCTTGG

And 4T1 cells were seeded on the well plate, and treated with different samples each other that contained PLNPs, SDNPs, Mϕ-NPs, Mϕ-SDNPs (10 μg/mL) at 37℃. After 4 h, cancer cells were treated with TGF-β (20ng/mL), except for non-treat group, for 16 h at 37℃, and cell lysates from each group were gained using RIPA buffer (Thermo Fisher Scientific, USA). The lysates were Homogenized, followed by ice incubation for 30 min, and lysates were centrifuged at 16,000 rpm at 4℃ for 30 min. The concentrations of supernatants were determined via BSA assay, and they were mixed with Laemmli buffer (5 mM dithiothreitol), boiled at 95℃ for 10 min, and the samples were loaded into 10% SDS-PAGE gels followed by electrophoresis at 60mV. After that, proteins in the agarose gel were transferred to a PVDF membrane (Millipore, USA) via Trans-Blot Turbo Transfer System (Bio-Rad, USA). Anti-E-cadherin, vimentin, β-actin antibodies, and anti-rabbit IgG antibody-HRP (Abcam, UK) were used for immunodetection.

### In vitro immunomodulation effect of Mϕ-SDNP

In order to analyze the repolarization of M2 macrophages, qRT-PCR analysis was performed. Bone-marrow-derived macrophages(BMDMs) were seeded on the well plate and treated with different samples containing SDNPs, MϕNPs, Mϕ-SDNPs (10 μg/mL) for 12 h at 37℃ to analyze M1/M2 marker on transcription level via qRT-PCR. And BMDM cells were treated with TGF-β (20ng/mL), except for the non-treat group, for 2 h at 37℃, and total RNA was isolated from BMDM cells via RNeasy mini kits (Qiagen, Germany), and cDNA was synthesized by using iScript cDNA synthesis kits (Bio-Rad, USA). The mRNA expression was normalized by mouse GAPDH expression, and the M1/M2 markers were calculated respectively by the ΔΔCt method.

**Table:** 

Target	Primer	Sequence (5’ – 3’)
cd206	Forward	CTGCAGATGGGTGGGTTATT
	Reverse	GGCATTGATGCTGCTGTTATG
Il-10	Forward	ACTGGCATGAGGATCAGCAG
	Reverse	CTCCTTGATTTCTGGGCCAT
arg1	Forward	AACACTCCCCTGACAACCAG
	Reverse	CCAGCAGGTAGCTGAAGGTC
fizz1	Forward	AGGATGCCAACTTTGAATAGGA
	Reverse	CGAGTAAGCACAGGCAGTT
cd86	Forward	GATTATCGGAGCGCCTTTCT
	Reverse	CCACACTGACTCTTCCATTCTT
il-6	Forward	ATCCAGTTGCCTTCTTGGGACTGA
	Reverse	TTGGATGGTCTTGGTCCTTAGCCA
tnf-α	Forward	CCTGTAGCCCACGTCGTAGC
	Reverse	AGCAATGACTCCAAAGTAGACC
inos	Forward	TCACCTTCGAGGGCAGCCGA
	Reverse	TCCGTGGCAAAGCGAGCCAG

### In vivo biodistribution of MϕNP

When the 4T1 tumor size reached about 200mm^3^, mice were intravenously injected with PLNP or MϕNP (particle dose: 25 mg/kg). Tumor-localized nanoparticles were monitored, and the fluorescence intensity of Cy5.5 was measured at 24 h post-injection by using FOBI.

### In vivo therapeutic efficacy study

Six to Eight weeks-old female Balb/c mice were injected with 2 × 10^5^ of 4T1 cells through subcutaneous injection in the mammary gland by gently penetrating the skin. When tumor size reached 50 mm^3^, mice were intravenously injected with treating nanoparticles (25 mg/kg) every 3 days for 4 times. And surgical resection and suture of the primary tumor were performed to confirm the efficacy of immunotherapy and improvement of survival rate by distant metastasis treatment. In the combination therapy experiments, anti-PD-1 antibody (Clone: RMP1-14, BioXcell) was diluted in PBS (1 mg/ml) and intravenously injected at a 5 mg/kg dose.

### Flow cytometric analysis of tumor immune cells

Mice were sacrificed, and the tumor was harvested and digested with collagenase for 30 min at 37 °C and filtered through cell strainer (100 μm pore size). After red blood cell lysis, all cells were washed three times with PBS and stained with antibodies for flow cytometry analysis. To evaluate intracellular cytokine levels, total cells were fixed and permeabilized by using BD Cytofix/Cytoperm™ Plus Kit (BD Bioscience, USA), and stained with anti-FoxP3, IFN-γ antibodies for flow cytometry analysis.

### Immunofluorescence imaging analysis

The harvested tumor samples were fixed and paraffin sectioned to 6 μm slices. Deparaffinized tumor sections were permeabilized with 0.1% Triton X-100 for 10 min. The tissues were washed with PBS and incubated with 1% BSA in PBS containing 0.1% Tween 20 (PBST) for 30 min to block the nonspecific binding of antibodies onto tumor cells. The sectioned samples were then stained with fluorescence-conjugated antibodies for Alexa 488-conjugated CD8 antibody (Santacruz, Dallas, USA), FITC conjugated CD206 antibody(biolegend), PerCP Cy5.5 conjugated CD86 antibody (Biolegend) and Alexa 647-conjugated granzyme B antibody (Santacruz) at 4 °C overnight in the dark condition. Cell nuclei were counterstained with DAPI with a final concentration of 300nM (Biolegend) and tumor tissues were mounted with Dako Fluorescence Mounting Medium. Fluorescence imaging was performed using AxioScan Z1 (Zeiss, Baden-Württemberg, Germany).

### Anti-metastasis efficacy of Mϕ-SDNP in 4T1 breast cancer lung metastasis model

Eight weeks-old Balb/c mice were injected with 1 × 10^5^ cells of 4T1 cell through tail vein to establish a metastatic tumor model. Mice were randomly divided into 5 groups for the different treatments. At day 13 post cell injection, mice were sacrificed and lung tissue was harvested to count metastatic nodules.

### H&E staining of lung metastasis tissue

Harvested lung tissue was fixed with 4% PFA for 2 days, and H&E staining was carried out upon external request at the experimental animal laboratory of Hanyang University and imaged.

All animal experiments were conducted according to the protocol approved by the Institutional Animal Care and Use Committee of Hanyang University, registered as 2021-0258 A.

### Schematic illustration design

all the schematic illustration was designed by using BioRender.

## Results

### Synthesis and Physicochemical characterization of MϕNP and Mϕ-SDNP

**Fig. 1:**
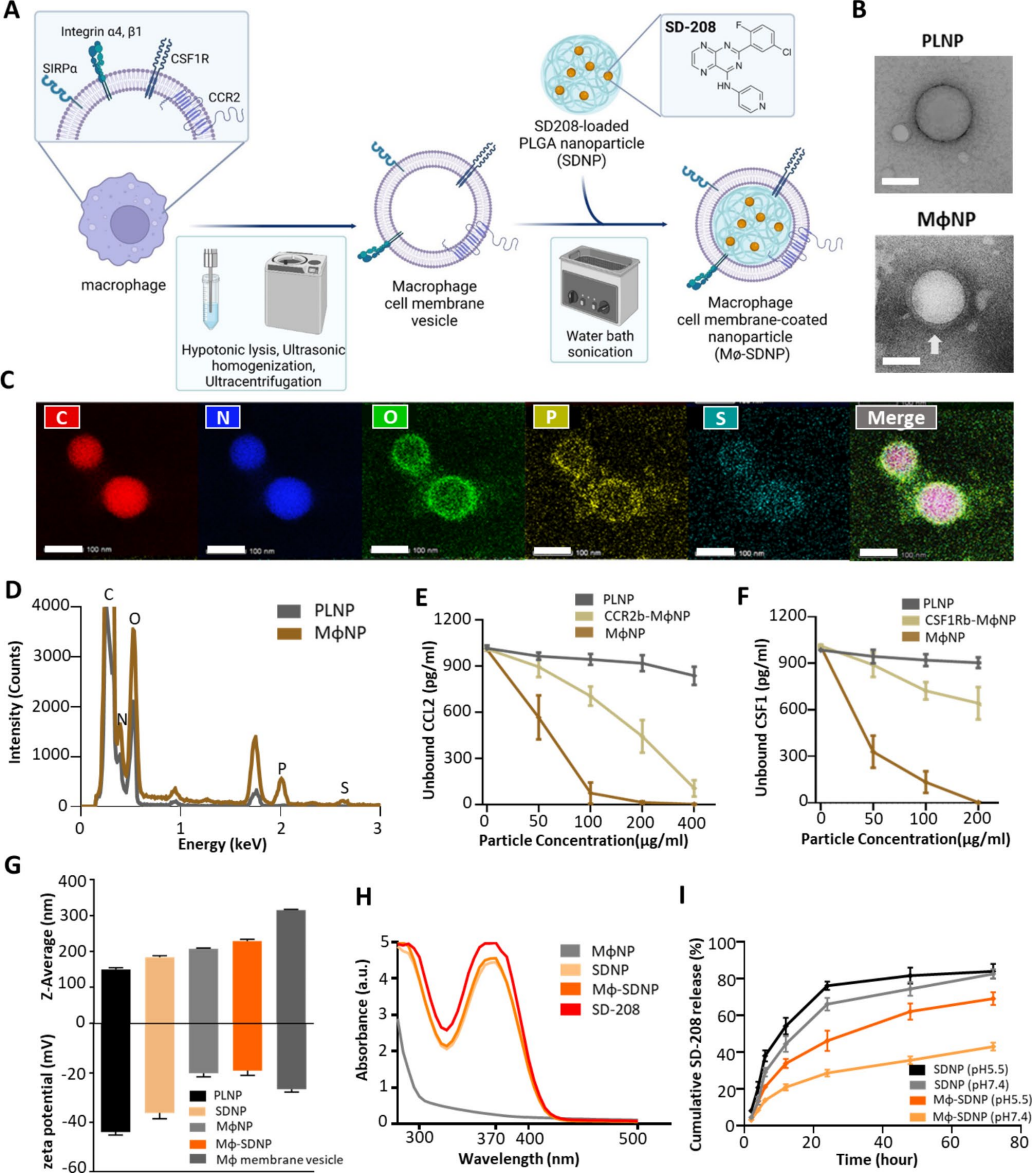
Synthesis and physicochemical characterization of MϕNP and Mϕ-SDNP. (**A**) Fabrication process of Mϕ-SDNP using conventional cell-membrane isolation and sonication. (**B**) Transmission electron microscopy (TEM) images of PLGA nanoparticles (PLNP) and macrophage-membrane-coated PLGA nanoparticles (MϕNP). Scale bars = 100 nm. (**C**) Energy-dispersive spectroscopy (EDS) elemental mapping analysis image of MϕNP. Scale bars = 100 nm. (**D**) EDS total intensity distribution of PLNP and MϕNP. E - **F**) Quantification of the protein scavenging effect of MϕNP by ELISA. CCR2 or CSF1R blocked MϕNP was represented to CCR2b- or CSF1Rb-MϕNP. (**E**) Unbound CCL2 level. (**F**) Unbound CSF1 level. (**G**) Hydrodynamic size and zeta potential analysis of SD-208-loaded macrophage membrane-coated nanoparticles (Mϕ-SDNP) by DLS. (**H**) The process of macrophage membrane coating does not affect the drug loading of nanoparticles. Spectrophotometric analysis of SD-208-loaded nanoparticles. (**I**) In vitro SD-208 release profiles obtained with a microplate reader at a wavelength of 370 nm

MϕNP and Mϕ-SDNP were synthesized employing the sonication technique from conventional membrane-coating methodologies. The core nanoparticles are poly(lactic-co-glycolic) acid (PLGA)-based and encapsulate SD-208, formulated via the O/W (oil-in-water) single emulsion method. Murine macrophage (Raw 264.7 cells) membranes were subsequently extracted, amalgamated with the nanoparticles, and sonicated to facilitate coating (Fig. 1A). To validate the effective encapsulation of the membrane on the nanoparticle surface, we performed transmission electron microscopy (TEM) and energy-dispersive X-ray spectroscopy (EDS) element mapping analysis (Fig. 1B-D). TEM imaging of the macrophage-membrane-coated nanoparticle (MϕNP) indicated a surface morphology that varied from the uncoated PLGA nanoparticle (PLNP), displaying characteristic spherical core-shell structures (Fig. 1B). Furthermore, EDS element mapping analysis substantiated the presence of plasma membrane component elements such as nitrogen (N), phosphorus (P), and sulfur (S) on the MϕNP surface (Fig. 1C), with the total intensity distribution differing from PLNP in respect to N, P, and S elements. MϕNPs showed N, P, S signals with much stronger intensity than PLNP (Fig. 1D). This suggests that, consistent with prior data, the MϕNPs are effectively coating the plasma membrane. The existence of surface proteins such as CCR2, CSF1R, SIRPα, and integrin α4 on the MϕNPs was detected through western blot assay (Fig. S1).

We postulated that MϕNPs bearing CCR2 and CSF1R on their surface could sequester CCL2 and CSF1, respectively. This was investigated by exposing CCL2 and CSF1 to varying concentrations of MϕNPs, CCR2 antibody-blocked MϕNPs (CCR2b-MϕNP), and CSF1R antibody-blocked MϕNPs (CSF1Rb-MϕNP). The residual CCL2 and CSF1 declined proportionally with the concentration of treated MϕNPs, while the antibody-blocked MϕNPs exhibited diminished protein scavenging capabilities (Fig. 1E, F). These findings suggest that MϕNPs retain the surface proteins and functional capacity of the macrophage membrane. Subsequently, we speculated that MϕNPs with SIRPα on their surface could disrupt the SIRPα-CD47 interaction between macrophages and cancer cells, hence activating macrophage phagocytic capacity. To this end, we applied MϕNPs and SIRPα-blocked MϕNPs to CFSE-stained cancer cells. After 4 h, macrophages (Raw 264.7 cells) were co-cultured to engulf these cancer cells. Flow cytometry analysis after incubation revealed enhanced phagocytic activity in macrophages co-cultured with MϕNP-treated cancer cells compared to those cultured with SIRPα-blocked MϕNPs or plain nanoparticles (Fig. S2). These results confirm that surface proteins on MϕNP can sequester CCL2 and CSF1 cytokines in the tumor microenvironment, thereby reinstating the recruitment of TAM. We also verified the enhancement of cancer cell phagocytosis by macrophages.

To capitalize on the synergistic potential of surface proteins on MϕNPs, we loaded the TGF-βR1 kinase inhibitor SD-208 within the nanoparticles and then coated them with the macrophage membrane (Mϕ-SDNP). The drug loading and encapsulation efficiencies of SDNP were optimized by modulating the weight ratios of SD-208 to PLGA, with the most efficient encapsulation and drug loading observed at a 10% ratio (Fig. S3). Hydrodynamic size and surface zeta potential of MϕNPs and Mϕ-SDNPs were analyzed using dynamic light scattering. As SD-208 was loaded or the macrophage membrane was coated, a slight increase in hydrodynamic size was noted (PLNP: 149.6 ± 5.12 nm, SDNP: 184.43 ± 4.15 nm, MϕNP: 207.4 ± 2.10 nm, and Mϕ-SDNP: 228.83 ± 5.09 nm). The surface zeta potentials of MϕNPs (-20.06 ± 1.52 mV) and Mϕ-SDNPs (-19.10 ± 1.87 mV) reflected values closer to the membrane vesicle (-26.57 ± 1.15 mV) than to PLNP (-43.93 ± 1.17mV) or MϕNP (-36.20 ± 2.30) (Fig. 1G). Consistent with prior studies, colloidal stability in serum persisted beyond 96 h following membrane coating (Fig. S4). Spectrophotometric analysis confirmed that nanoparticle synthesis and macrophage membrane coating processes did not impact the stability of SD-208 (Fig. 1H). Additionally, the drug release study displayed a prolonged and sustained release pattern for membrane-coated nanoparticles, with 82.58 ± 2.50% to 43.03 ± 2.18% of SD-208 at pH 7.4, and 83.96 ± 3.99% to 69.12 ± 3.4% of SD-208 at pH 5.5 released after 72 h of incubation (Fig. 1I).

**Fig. 2:**
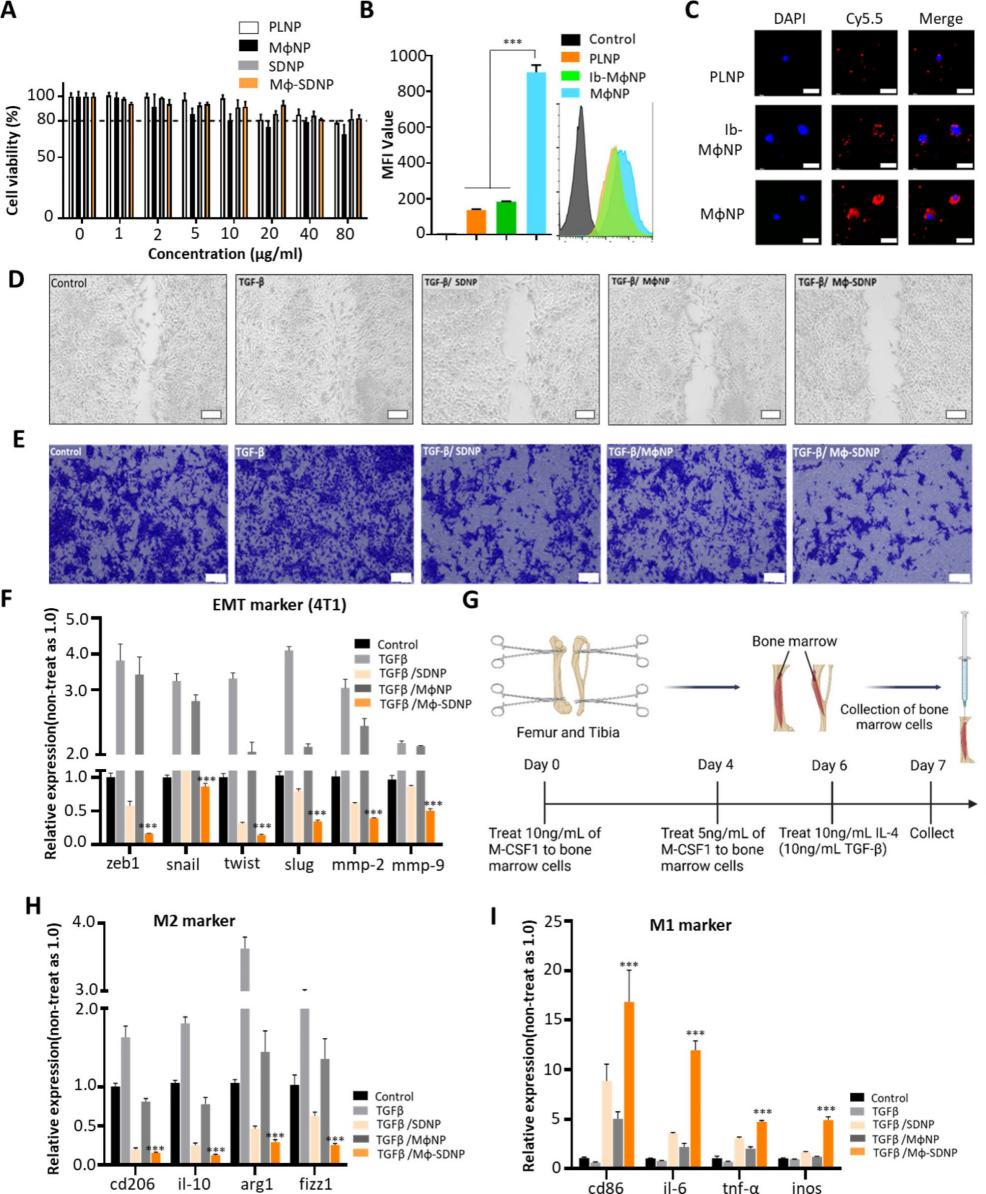
Inhibition of TGF-β-mediated cancer cell migration and macrophage polarization by Mϕ-SDNP. (**A**) Viability of 4T1 cells after treatment with PLNP, SDNP, MϕNP, and Mϕ-SDNP as determined using MTT assay. (**B**) Flow cytometric analysis for cellular uptake post Cy5.5-loaded MϕNP treatment. ***p < 0.001. Statical significance was calculated with Student’s t-test (n = 3). (**C**) Representative confocal fluorescence microscope images. Nuclei were stained with DAPI. Scale bars = 15 μm. PLGA nanoparticles (PLNP), Integrin α4-blocked macrophage membrane-coated nanoparticle (Ib-MϕNP), and macrophage membrane-coated nanoparticles (MϕNP) were treated. D - **E**) SD-208-loaded MϕNP (Mϕ-SDNP) significantly reduced TGF-β-mediated cancer cell invasion. (**D**) Wound healing assay. 10 μg/ml of each nanoparticle was applied, and TGF-β was treated at a concentration of 20ng/ml. Scale bars = 100 μm. (**E**) Transendothelial migration assay. The HUVEC cell monolayer migrated cancer cells were stained with crystal violet. The relatively small dot represents migrated cancer cell. Scale bars = 100 μm. (**F**) EMT marker gene expression analysis. The mRNA expression was normalized by mouse GAPDH expression. (**G**) Schematic representation of the BMDM isolation and M2-type differentiation process. **H**-**I**) Relative mRNA expression analysis of (**H**) M2 macrophage marker and (**I**) M1 macrophage marker. The mRNA expression was normalized by mouse GAPDH expression. ***p < 0.001. Statistical significance was calculated with Student’s t-test

The ideal treatment concentration of MϕNPs and Mϕ-SDNPs for in vitro experiments was determined through cell viability testing to evaluate the cytotoxicity of the nanoparticles. A concentration of 10–20 μg/ml was established as the optimal concentration for providing an effective amount of the encapsulated drug and maintaining the functionality of membrane proteins without compromising cell viability (Fig. 2A). Furthermore, we compared nanoparticle internalization in 4T1 cancer cells using Cy5.5-loaded PLNPs, MϕNPs, and integrin α4-blocked MϕNPs (Ib-MϕNP) through flow cytometry and confocal microscopy imaging. MϕNPs significantly enhanced internalization efficiency, with approximately 6.63-fold and 4.91-fold higher mean fluorescence intensity (MFI) values than PLNPs and Ib-MϕNPs, respectively (Fig. 2B). Similar results were observed through fluorescence microscopy imaging (Fig. 2C). These findings confirm that coating with a macrophage membrane enhances nanoparticle cellular internalization, promoting efficient intracellular drug delivery. It also underscores the integral role that the integrin protein plays in nanoparticle uptake by 4T1 breast cancer cells.

### Dual inhibition of TGF-β-mediated epithelial-to-mesenchymal transition (EMT) of cancer cells and M2 polarization of macrophages by Mϕ-SDNP

TGF-β plays a crucial role in inducing the EMT in cancer cells, which contributes to metastasis. We hypothesized that Mϕ-SDNP could inhibit the TGF-β-mediated EMT, subsequently impeding cancer cell invasiveness. To evaluate this, we utilized the wound-healing assay to assess the migration pattern of cancer cells. In TGF-β-treated 4T1 cells, rapid migration and notable scratch closure were observed. In contrast, the SD-208-loaded nanoparticle-treated groups (SDNP and Mϕ-SDNP) displayed minimal scratch recovery despite TGF-β treatment. Particularly in 4T1 cells treated with Mϕ-SDNP, the scratch remained largely unfilled, demonstrating significant inhibition of active 4T1 cell migration by Mϕ-SDNP (Fig. 2D).

In addition to the wound-healing assay, a transendothelial migration assay was conducted to evaluate 4T1 cell extravasation. Post nanoparticle treatment, 4T1 cell groups were placed into the upper chamber of a co-culture plate, covered with a monolayer of human umbilical vein endothelial cells (HUVECs). TGF-β-treated cells displayed significantly increased migration compared to untreated cells, while minimal migration was observed in Mϕ-SDNP-treated cells (Fig. 2E). The combined results of the wound-healing and TEM assays suggest that SD-208-loaded nanoparticles effectively block the TGF-β-induced invasiveness of cancer cells.

To validate the above observations, we employed qRT-PCR and western blot assays to measure EMT marker expression at mRNA and protein levels, respectively. TGF-β treatment upregulated all examined mRNA levels of EMT markers, including zeb1, snail, twist, slug, mmp-2, and mmp-9. However, these increases were significantly reduced following Mϕ-SDNP treatment (Fig. 2F). Additionally, while TGF-β treatment reduced E-cadherin expression, Mϕ-SDNP treatment maintained its expression level (Fig. S5). These results confirm that Mϕ-SDNP is efficiently uptaken by cancer cells, effectively suppressing TGF-β receptor signaling, and subsequently, the EMT-mediated invasiveness of cancer cells.

TGF-β is also implicated in macrophage polarization within the tumor environment, promoting an immunosuppressive milieu by inducing M2-type macrophage differentiation. We assessed whether Mϕ-SDNP can counter this M2-type macrophage differentiation. Bone marrow-derived macrophages (BMDMs) were harvested from mice and treated with nanoparticles after M2 polarization. M1 and M2 macrophage markers were subsequently analyzed at the mRNA level (Fig. 2H, I). TGF-β treatment stimulated M2 differentiation, whereas Mϕ-SDNP treatment significantly reduced the expression of M2 genes such as cd206, il-10, arg1, and fizz1 (Fig. 2H). Additionally, Mϕ-SDNP treatment significantly increased the expression of M1 genes including cd86, il-6, tnf-α, and inos (Fig. 2I). This demonstrates that Mϕ-SDNP effectively reverses TGF-β-mediated M2 macrophage differentiation, thereby modulating the tumor microenvironment.

### In vivo biodistribution and tumor targeting of MϕNP in orthotopic 4T1 breast cancer model

**Fig. 3:**
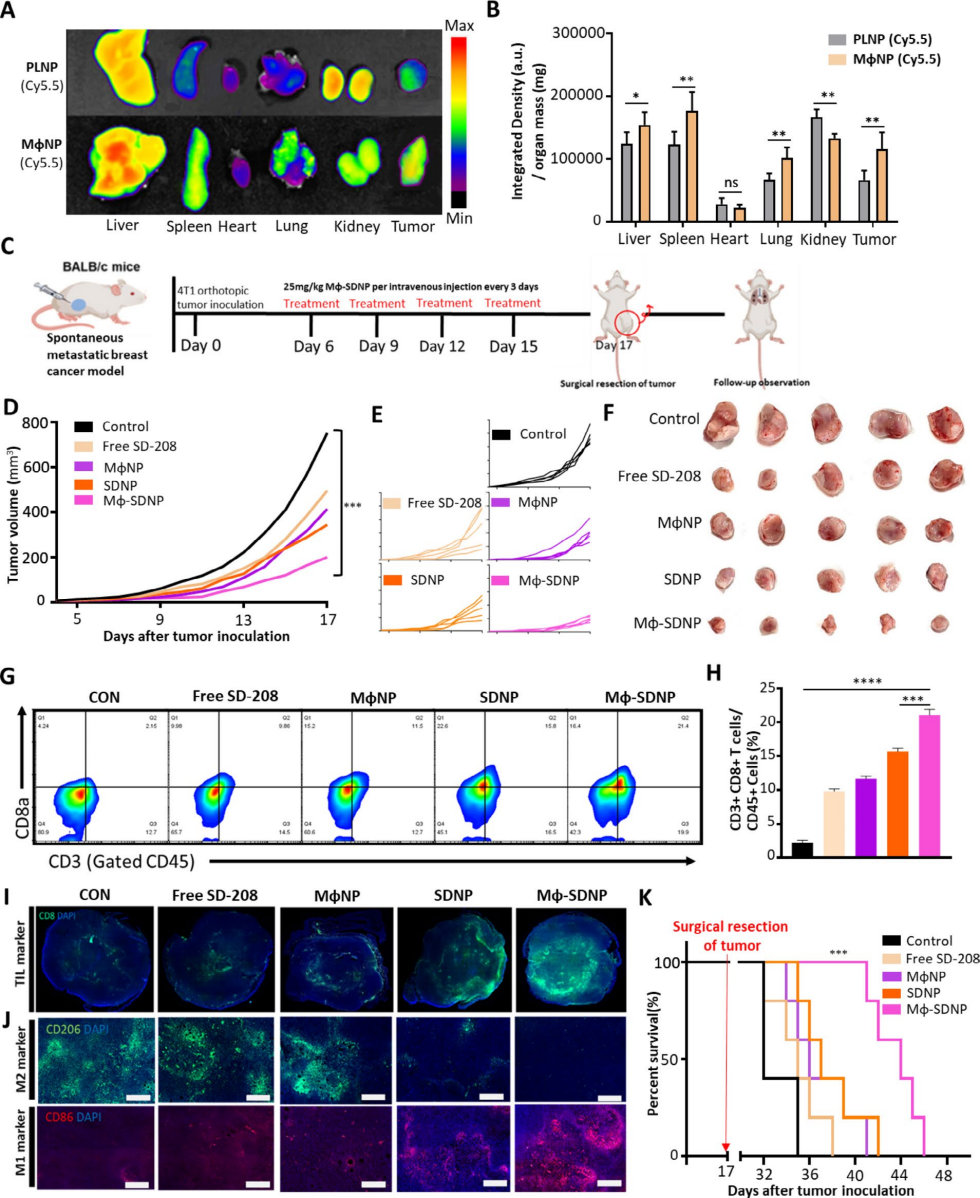
In vivo anti-cancer and anti-metastasis efficacy of Mϕ-SDNP in orthotopic 4T1 breast cancer model. (**A**) Representative ex vivo images and (**B**) quantification data of fluorescence signals in the major organs and tumors of mice 24 h after intravenous injection of Cy5.5-loaded PLNPs and MϕNPs. *p < 0.05, **p < 0.01 Statical significance was calculated with Student’s t-test (n = 3). (**C**) Schematic illustration of treatment schedules for verifying anti-tumoral efficacies of Mϕ-SDNP. (**D**) Average growth profiles of tumors (n = 5). (**E**) Growth profiles of each tumor (n = 5). (**F**) Obtained tumors after surgical resection on day 17. **G**-**H**) Flow cytometric analysis of the primary tumor for verifying immunomodulation efficacies of Mϕ-SDNP. **I**) Representative immunofluorescence images of tumor-infiltrating lymphocyte staining. Green = CD8, Blue = DAPI. **J**) Immunofluorescence image of M1 and M2 macrophage staining. Green = CD206, Red = CD86, Blue = DAPI. **K**) Survival profiles after treatment with Mϕ-SDNP and surgical resection (n = 5)

The biodistribution and tumor-targeting abilities of MϕNPs were assessed in an orthotopic 4T1 breast cancer model, a model known for spontaneous lung metastasis. Cy5.5-loaded PLNPs or MϕNPs were administered intravenously to mice bearing 4T1 orthotopic tumors. After 24 h post-injection, major organs and tumors were isolated for evaluation. The Cy5.5-loaded MϕNPs displayed a remarkably improved primary and metastatic tumor-targeting capabilities compared to PLNPs (Fig. 3A, B). Additionally, the study utilized immunofluorescence staining to assess if nanoparticles were delivered to tumor-associated macrophages within tumor tissues. The results confirmed that the nanoparticles were efficiently delivered to the tumor-related macrophages and cancer cells (Fig. S6).

Despite significant accumulation of nanoparticles in the liver and kidneys observed in Fig. 3A and B, enzyme levels indicating liver and kidney damage remained low (Fig. S7), suggesting the absence of Mϕ-SDNP toxicity. This could imply a temporary transit of nanoparticles through reticuloendothelial organs.

### In vivo anti-cancer and anti-metastasis efficacy of Mϕ-SDNPs

The in vivo cancer immunotherapeutic efficacy of Mϕ-SDNPs was assessed using Balb/c mice bearing orthotopic 4T1 tumors. Mice were treated with 25 mg/kg of Mϕ-SDNPs via intravenous injection every three days. A surgical resection of the primary tumor was performed on day 17 to evaluate the potential prolonged survival rate due to the anti-metastatic efficacy of Mϕ-SDNPs (Fig. 3C). The Mϕ-SDNP treatment noticeably delayed primary tumor growth in comparison with other groups (Fig. 3D-F). There were no significant deviations in body weight across the treatment groups, indicating that the nanoparticles did not induce significant in vivo toxicity (Fig. S8). To verify the immunomodulating mechanism of Mϕ-SDNP, immune cells in the primary tumor tissue were analyzed. Flow cytometry revealed a significant increase in the proportion of CD8 + cytotoxic T cells (CTLs) in the tumor of the Mϕ-SDNP-treated group (Fig. 3G). In fact, the CTL ratio in the tumor from the Mϕ-SDNP-treated group was 9.3 times higher than in the control group (Fig. 3H). This was consistent with immunofluorescence images that showed a higher influx of CTLs into tumor tissue following Mϕ-SDNP treatment in comparison with the control group (Fig. 3I). Furthermore, effective delivery of Mϕ-SDNPs to macrophages in the tumor was confirmed to induce the repolarization of tumor-associated macrophages via TGF-β inhibition. Through an immunofluorescence staining, clear distinction of M1 polarization compared to the control group was revealed (Fig. 3J). Most of the treated animals that received surgical resection did not exhibit tumor regrowth, but succumbed to metastasis to secondary sites. The Mϕ-SDNP-treated group displayed notably better survival rates than other control groups. This suggests that the Mϕ-SDNP provided a significant impact in reducing the growth and spread of the tumor (Fig. 3K).

**Fig. 4:**
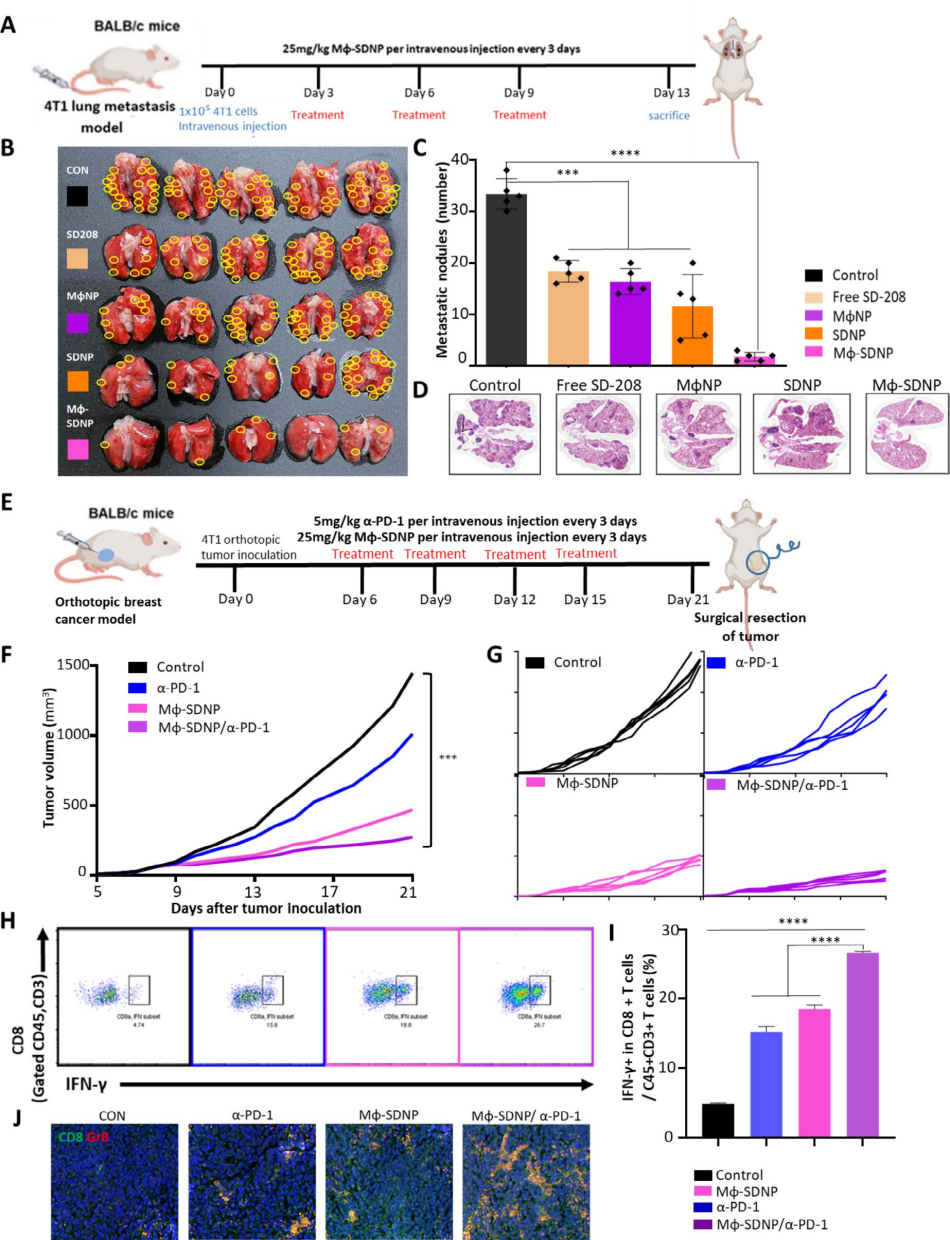
Anti-metastatic and synergistic anti-tumoral efficacy of Mϕ-SDNP and immune checkpoint inhibitor. (**A**) Schematic illustration of treatment schedules for verifying anti-metastasis ability. (**B**) Ex vivo lung image representing the anti-metastasis ability of Mϕ-SDNP. (**C**) Comparison of metastatic nodule number. ***p < 0.001, ****p < 0.0001 Statistical significance was calculated with Student’s t-test (n = 5). (**D**) Representative H&E staining image of lung metastasis. (**E**) Schematic illustration of treatment schedules for verifying the synergistic effect of combination treatment with anti-PD-1 antibody. **F**-**G**) Tumor growth profile until surgical resection of the tumor on day 21. **H**-**I**) Verification of T cell activation with flow cytometric analysis of IFN-γ^+^ CTL recruitment inside the tumor tissue. **J**) Representative immunofluorescence image for verifying the activation of granzyme B^+^ cytotoxic T cells

The anti-metastatic effect of Mϕ-SDNP was confirmed by intravenously injecting 4T1 cells into Balb/c mice, mimicking aggressive breast cancer metastasis. Mice were treated with Mϕ-SDNPs every three days and sacrificed on day 13 (Fig. 4A). As a result, lung metastasis was significantly reduced in both the SD-208 and macrophage membrane-coated nanoparticles groups compared to the non-treated control group. Specifically, the Mϕ-SDNP-treated group had the lowest number of metastatic nodules in the lungs (Fig. 4B). The non-treated group had 33 metastatic nodules on the lung surface, in contrast to an average of two nodules in the Mϕ-SDNP group, validating significant metastasis inhibition by Mϕ-SDNP (Fig. 4C). H&E staining analysis further confirmed that Mϕ-SDNP has a strong inhibitory effect on lung metastasis (Fig. 4D). The in vivo luciferase assay also demonstrated the robust anti-metastatic effect of Mϕ-SDNP treatment in 4T1-Luc2-bearing mice (Fig. S9).

### Efficacy of combination cancer immunotherapy with Mϕ-SDNP and immune checkpoint inhibitor

Given the demonstrated ability of Mϕ-SDNP treatment to robustly recruit T cells into primary tumors, the possibility of increased response rates to immune checkpoint inhibitors was explored. To investigate the potential synergistic effect of Mϕ-SDNP with a conventional immune checkpoint inhibitor, orthotopic 4T1 tumor-bearing Balb/c mice were treated with 5 mg/kg of an anti-PD-1 antibody (α-PD-1) and 25 mg/kg of Mϕ-SDNP via intravenous injection every three days. Primary tumors were harvested on day 21 (Fig. 4E). The α-PD-1 treatment alone had only a slight inhibitory effect on tumor growth. However, the combination treatment of α-PD-1 and Mϕ-SDNP significantly inhibited tumor growth. Compared to the sole α-PD-1 treatment, the combination treatment of α-PD-1 with Mϕ-SDNP resulted in about 3.7-fold smaller tumor volumes on day 21 (Fig. 4F, G).

To determine whether the T cells recruited into the tumor tissue were effectively eradicating cancer cells due to the synergistic effect with the immune checkpoint inhibitor, CD8 + CTLs within the primary tumor tissue were analyzed. Flow cytometric analysis revealed a significant increase in the number of CD8 + CTLs when α-PD-1 was administered in combination with Mϕ-SDNP, compared to α-PD-1 alone. A significant increase in activated IFN-γ + CTL was also observed (Fig. 4F). The proportion of IFN-γ-secreting CTLs in tumor tissue increased by about 1.7-fold from around 15.6% with α-PD-1 alone to 26.7% when administered in combination with Mϕ-SDNP (Fig. 4I). Furthermore, both the Mϕ-SDNP treatment and combination therapy with the immune checkpoint inhibitor significantly reduced the populations of CD4 + Foxp3 + regulatory T cells (Fig. S10). Enhanced anti-cancer activity of cytotoxic T cells was again validated in immunofluorescence images. Consistent with previous in vivo results, increased CD8 + CTL infiltration was observed following Mϕ-SDNP treatment, and granzyme B activity of CTL was measured to validate the synergistic effect of combining Mϕ-SDNP with α-PD-1 (Fig. 4J). Upon surgical tumor resection, the survival rate of mice treated with the combination of Mϕ-SDNP and the immune checkpoint inhibitor was significantly longer compared to other groups (Fig. S11).

**Scheme 1:**
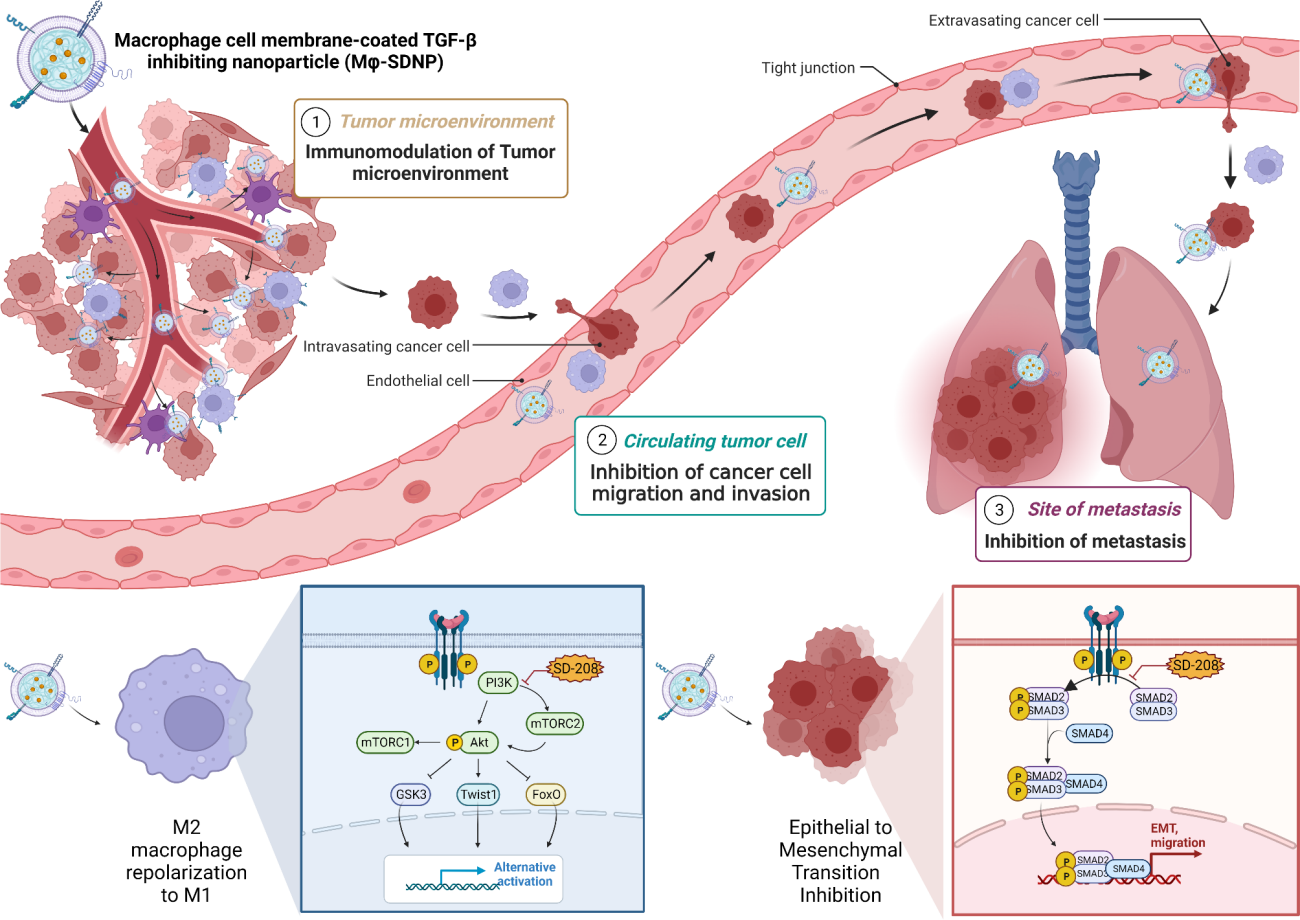
Schematic illustration of macrophage-membrane-coated and a TGF-βR1 kinase inhibitor (SD-208)-loaded nanoparticle (Mϕ-SDNP) for anti-cancer and anti-metastatic immunotherapy Mϕ-SDNP effectively targets cancer cells and tumor-associated macrophages (TAMs). Dual TGF-β signaling inhibition by Mϕ-SDNP suppresses cancer cell metastasis and reprograms TAMs for immunoboosting

## Discussion

Overcoming the low response rate of cancer immunotherapy necessitates a broad understanding of immune evasion mechanisms within the tumor microenvironment [16]. Among the various cells in the tumor microenvironment, tumor-associated macrophages (TAMs) help cancer cells grow and metastasize and create an immunosuppressive area that reduces the response rate of anti-cancer immunotherapy [18]. Based on these points, we developed effective treatments with two main points to overcome the immunosuppressive environments. First, to develop a carrier that can simultaneously deliver cargo to cancer cells and tumor-related macrophages. And second, to induce effective anti-cancer immunotherapy for cancer cells and TAMs.

Transforming growth factor-β (TGF-β) is known to reduce responsiveness to cancer immunotherapy by promoting M2-type differentiation of macrophages and assisting cancer cells in metastasis through induction of the epithelial-to-mesenchymal transition (EMT) in cancer cells [23–25]. Accordingly, we explored a drug called SD-208, which inhibits the TGF-β signaling pathway and can be encapsulated on a hydrophobic core of PLGA nanoparticles. In addition, to effectively deliver the drug inside the tumor and to the site of metastasis, a macrophage cell membrane was extracted and coated around the surface of the SD-208-loaded PLGA nanoparticles (Mϕ-SDNPs) to mimic the function of macrophages inside the tumor microenvironment.

These enhanced tumor-targeting abilities of MϕNPs can be attributed to two mechanisms. First, the leaky tumor vasculature may allow nanoparticles to infiltrate and accumulate in the tumor tissue, known as the enhanced permeation and retention (EPR) effect. Second, the various surface proteins derived from macrophages may enable MϕNPs to cluster around the tumor microenvironment. Prior studies have indicated that integrin α4 and β1 on macrophages could interact with vascular cell adhesion molecule-1 (VCAM-1) on cancer cells [26]. Moreover, CCR2 and CSF1R surface proteins of MϕNP may interact with CCL2 and CSF1, respectively, which are known for recruiting monocytes and macrophages into the tumor tissue.

The metastasis-tracking ability of MϕNPs is assumed to be driven by their surface proteins. The CCL2-CCR2 and CSF1-CSF1R axis have been reported to recruit monocytes and metastasis-associated macrophages (MAMs) [27, 28]. The CCL2 and CSF1 metastatic sites might draw MϕNPs into the metastatic tumor regions. Given previous reports indicating VCAM-1 of circulating cancer cells transmitting survival signals in breast cancer cells invading the lung, the integrin α4 on MϕNPs could potentially interact with VCAM-1 of circulating cancer cells, aiding their migration into the metastatic site [20]. Based on this, Mϕ-SDNPs were effectively accumulated in tumor tissue and site of metastasis, and effective anti-tumor/metastasis activity was verified.

The therapeutic mechanisms of Mϕ-SDNPs that we emphasize in this research are as follows : Firstly, Mϕ-SDNPs can specifically target the tumor site and metastasis site owing to the inherent characteristics of proteins on the particle surface derived from macrophages, such as integrin – VCAM1 binding. Secondly, Mϕ-SDNPs can scavenge CCL2 and CSF1 in the tumor site and exploit the SIRPα-CD47 interaction to remodel the tumor microenvironment. Lastly, Mϕ-SDNPs can directly inhibit TGF-β-driven cancer cell invasion and M2-type macrophage polarization. These mechanisms enable TGF-β signaling inhibitor (SD-208)-loaded Mϕ-SDNPs to inhibit cancer metastasis and remodel the immunosuppressive tumor microenvironment (or “cold” tumor) into an immunostimulatory tumor microenvironment (or “hot” tumor) by blocking M2-type macrophage differentiation (Scheme 1). As a result, the population of CTLs in the tumor tissue was significantly increased following treatment with Mϕ-SDNPs.

Although we only studied orthotopic breast cancer and breast cancer lung metastasis models, Clinically, our anti-cancer effect of Mϕ-SDNPs can be accompanied by a wide range of anti-cancer immunotherapy. Mϕ-SDNPs showed an effective anti-tumor effect even when administered alone, but since they showed a dramatic tumor-killing effect when administered in combination with immune checkpoint inhibitors, research to improve the response rate of immune checkpoint inhibitors may also contribute. This point suggests that it can be used for immunotherapeutics at various sites from the primary tumor site to the site of metastasis, and indicates that it can be applied to various patient groups from stage 1 to stage 3 ~ 4 cancer patients.

## Conclusion

Macrophage membrane-coated nanoparticles loaded with a TGF-βR1 kinase inhibitor(Mϕ-SDNP) significantly improved responsiveness to immune checkpoint inhibitors, demonstrating a robust anti-cancer effect in conjunction with anti-PD-1 antibodies, which was resulted from inhibition of cancer metastasis, blockade of M2-type macrophage differentiation, and augmentation of the population of cytotoxic T lymphocytes (CTLs) in the tumor tissue. These findings highlight the potential of Mϕ-SDNP as a potent anti-cancer immunotherapy, especially in combination with conventional immune checkpoint inhibitors. Consequently, our findings suggest a new immunotherapeutic-booster approach to comprehensive anti-cancer immunotherapy for a wide range of cancer patients.

### Electronic supplementary material

Below is the link to the electronic supplementary material.

**Supplementary Material 1: Fig. S1.** Western blot analysis for membrane protein expression on MϕNP. MϕNP represented similar expression patterns with a macrophage membrane (Mϕ mem). **Fig. S2.** Phagocytic activity of macrophages to CFSE-stained 4T1 cells treated with MϕNP. CFSE-stained 4T1 cells and nanoparticle-treated macrophages were directly co-cultured. CFSE (-) area represented nonphagocytic macrophages. PLNP-treated group and SIRPα-blocked MϕNP-treated group showed the distinct distribution of each cells. On the other hand, the MϕNP-treated group showed a decreased CFSE(-) population, which means enhanced phagocytosis toward 4T1 cells. **Fig. S3.** Optimization of w/w ratio between SD-208 and PLGA in SD-208-loaded PLGA nanoparticle (SDNP) preparation process. Drug loading and encapsulation efficiency of SD-208 were analyzed (n = 3). The w/w ratio between SD-208 and PLGA was optimized at 10%. **Fig. S4.** Colloidal stability of Mϕ-SDNP in 50% serum, as evaluated by DLS (n = 3). The hydrodynamic size of Mϕ-SDNP remained stable up to 4 days. **Fig. S5.** Western blot images demonstrating inhibition of TGF-β-mediated epithelial-to-mesenchymal transition (EMT) with SD-208 loaded nanoparticles. Treatment with SDNP and Mϕ-SDNP to 4T1 cells recovered E-cadherin expression level, which was reduced by TGF-β. In addition, the expression level of vimentin, a mesenchymal cell marker, was decreased. **Fig. S6.** Immunostained tumor image demonstrating tumor-associated macrophage-targeting ability of MϕNP. Green signals represent tumor-associated macrophages (F4/80+), red signals represent Cy5.5-loaded MϕNP. It was shown that the MϕNPs penetrating inside the tumor tissue were well delivered to macrophages inside the tumor microenvironment. **Fig. S7.** In vivo toxicity evaluation of Mϕ-SDNP. Toxicity was analyzed by measuring the levels of enzymes reflecting the functions of the liver and kidneys, such as aspartate aminotransferase (AST), alanine aminotransferase (ALT), blood urea nitrogen (BUN), and creatinine (CREA) in the plasma of 4T1 tumor-bearing mice on the day of last injection. Data represent mean ± SD. ns = not significant difference. Statical analysis was followed by student’s T test (n = 3). **Fig. S8.** Body weight profiles (n = 5). Data represent mean ± SD. ns = not significant difference. Statical analysis was followed by two-way ANOVA with Boneferroni post-tests (n = 5). **Fig. S9.** In vivo luciferase imaging for verifying anti-metastasis efficacy. The luminescence signal represents the luciferase signal from tail-vein-injected 4T1-luc2 cells. Luciferin was injected with 150mg/kg concentration. Intravenously injected 4T1-luc2 cells showed accumulation in the lungs 10 days after inoculation, and each nanoparticle-administered group showed a decreased accumulation of cells in the lungs. **Fig. S10.** Flow cytometric analysis of CD4 + Foxp3 + regulatory T cell population. The distribution of regulatory T cells within primary tumor tissue showed the lowest pattern in the combination treatment group with anti-PD-1 antibodies. Statical analysis was calculated by student’s t-test. ****p < 0.001. **Fig. S11.** In vivo anti-metastatic survival rate profile in combination therapy with anti-PD-1 antibody. After the first surgical resection of tumor tissue on day 17, it was confirmed that the response rate of the immune checkpoint inhibitor improved when nanoparticles and immune checkpoint inhibitors were administered together, resulting in a synergy effect

## Data Availability

The datasets used and/or analyzed during the current study are available from the corresponding author on reasonable request.

## Abbreviations

BMDMs: Bone marrow-derived macrophages
CCL2: Chemokine (C-C motif) ligand 2
CCR2: C-C chemokine receptor type 2
CSF1: Colony stimulating factor 1
CSF1R: Colony stimulating factor 1 receptor
CTLs: Cytotoxic T lymphocytes
EDS: Energy-dispersive spectroscopy
EMT: Epithelial to-mesenchymal transition
HUVECs: Human umbilical vein endothelial cells
MAMs: Metastasis associated macrophages
PLGA: Poly(lactic-co-glycolic) acid
SIRPα: Signal regulatory protein α
TAMs: Tumor associated macrophages
TEM: Transmission electron microscopy
TGF-β: Transforming growth factor β
TME: Tumor microenvironment
VCAM-1: Vascular cell adhesion molecule 1
