# Supplementary material for: Engineering TGF-β inhibitor-encapsulated macrophage-inspired multi-functional nanoparticles for combination cancer immunotherapy
Source: Biomater Res. 2023 Dec 18;27:136. doi: 10.1186/s40824-023-00470-y (PMC10729390; doi:10.1186/s40824-023-00470-y)
Supplement: Supplementary file 1 — Supplementary Material 1: Fig. S1. Western blot analysis for membrane protein expression on MϕNP. MϕNP represented similar expression patterns with a macrophage membrane (Mϕ mem). Fig. S2. Phagocytic activity of macrophages to CFSE-stained 4T1 cells treated with MϕNP. CFSE-stained 4T1 cells and nanoparticle-treated macrophages were directly co-cultured. CFSE (-) area represented nonphagocytic macrophages. PLNP-treated group and SIRPα-blocked MϕNP-treated group showed the distinct distribution of each cells. On the other hand, the MϕNP-treated group showed a decreased CFSE(-) population, which means enhanced phagocytosis toward 4T1 cells. Fig. S3. Optimization of w/w ratio between SD-208 and PLGA in SD-208-loaded PLGA nanoparticle (SDNP) preparation process. Drug loading and encapsulation efficiency of SD-208 were analyzed (n = 3). The w/w ratio between SD-208 and PLGA was optimized at 10%. Fig. S4. Colloidal stability of Mϕ-SDNP in 50% serum, as evaluated by DLS (n = 3). The hydrodynamic size of Mϕ-SDNP remained stable up to 4 days. Fig. S5. Western blot images demonstrating inhibition of TGF-β-mediated epithelial-to-mesenchymal transition (EMT) with SD-208 loaded nanoparticles. Treatment with SDNP and Mϕ-SDNP to 4T1 cells recovered E-cadherin expression level, which was reduced by TGF-β. In addition, the expression level of vimentin, a mesenchymal cell marker, was decreased. Fig. S6. Immunostained tumor image demonstrating tumor-associated macrophage-targeting ability of MϕNP. Green signals represent tumor-associated macrophages (F4/80+), red signals represent Cy5.5-loaded MϕNP. It was shown that the MϕNPs penetrating inside the tumor tissue were well delivered to macrophages inside the tumor microenvironment. Fig. S7. In vivo toxicity evaluation of Mϕ-SDNP. Toxicity was analyzed by measuring the levels of enzymes reflecting the functions of the liver and kidneys, such as aspartate aminotransferase (AST), alanine aminotransferase (ALT), blood urea nitrogen (BUN), a [file 40824_2023_470_MOESM1_ESM.docx]

Supporting Information

**Engineering TGF-β Inhibitor-encapsulated Macrophage-inspired Multi-functional Nanoparticles for Combination Cancer Immunotherapy**

Jaehyun Kim^1^, Minjeong Kim^1^, Seok-Beom Yong^2^, Heesoo Han^1^, Seyoung Kang^1^, Shayan F. Lahiji^1,4^, Sangjin Kim^1^, Juhyeong Hong^1^, Yuha seo^1^, and Yong-Hee Kim^1,3,4^*

^1^ Department of Bioengineering, Institute for Bioengineering and Biopharmaceutical Research

Hanyang University, 04763, Seoul, Republic of Korea

^2^ Nucleic Acid Therapeutics Research Center, Korea Research Institute of Bioscience and Biotechnology (KRIBB), Chungcheongbuk-do 28116, Republic of Korea

^3^ Institute for Bioengineering and Biopharmaceutical Research (IBBR), Hanyang University, 04763, Seoul, Republic of Korea

^4^ Cursus Bio Inc. Icure Tower, Gangnam-gu, Seoul 06170, Republic of Korea

*Correspondence: Yong-Hee Kim, Department of Bioengineering, Hanyang University, Seoul, Republic of Korea

E-mail: [yongheekim@hanyang.ac.kr](mailto:yongheekim@hanyang.ac.kr)

**Supporting Figures**


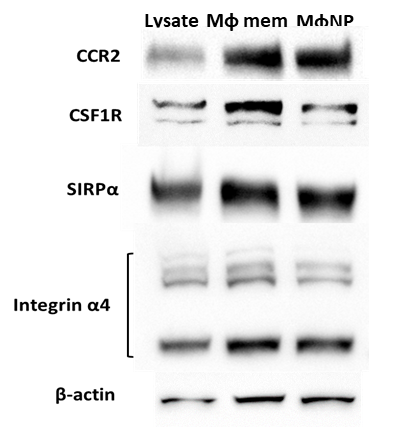


**Fig. S1.** Western blot analysis for membrane protein expression on MϕNP. MϕNP represented similar expression patterns with a macrophage membrane (Mϕ mem).

**
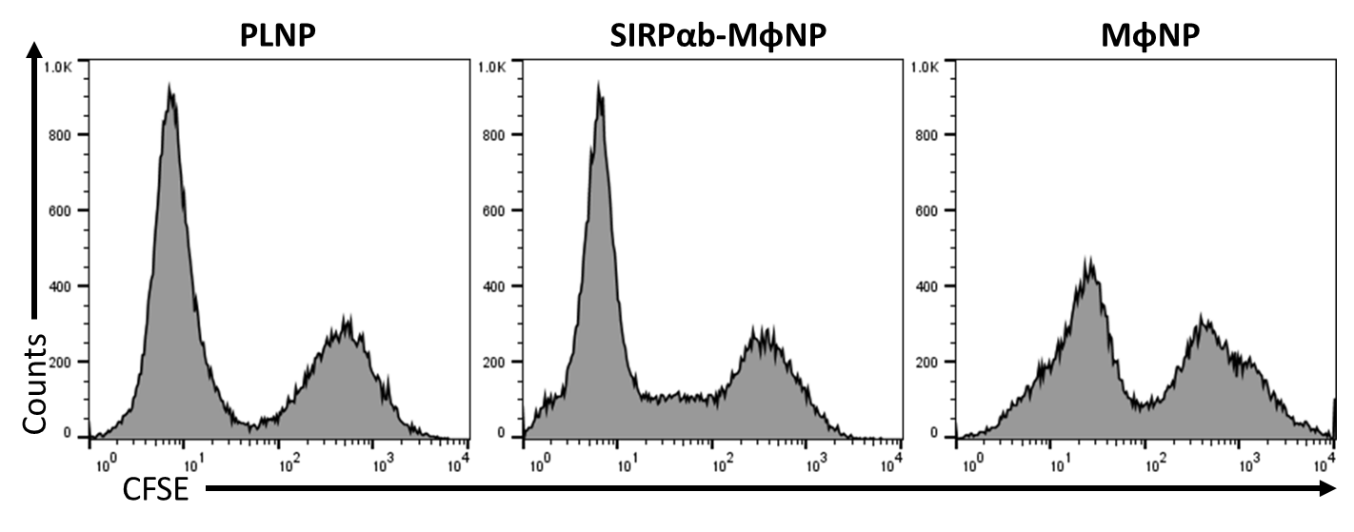
**

**Fig. S2.** Phagocytic activity of macrophages to CFSE-stained 4T1 cells treated with MϕNP. CFSE-stained 4T1 cells and nanoparticle-treated macrophages were directly co-cultured. CFSE (-) area represented nonphagocytic macrophages. PLNP-treated group and SIRPα-blocked MϕNP-treated group showed the distinct distribution of each cells. On the other hand, the MϕNP-treated group showed a decreased CFSE(-) population, which means enhanced phagocytosis toward 4T1 cells.

**Fig. S3.** Optimization of w/w ratio between SD-208 and PLGA in SD-208-loaded PLGA nanoparticle (SDNP) preparation process. Drug loading and encapsulation efficiency of SD-208 were analyzed (n=3). The w/w ratio between SD-208 and PLGA was optimized at 10%.

**Fig. S4.** Colloidal stability of Mϕ-SDNP in 50% serum, as evaluated by DLS (n=3). The hydrodynamic size of Mϕ-SDNP remained stable up to 4 days.

**
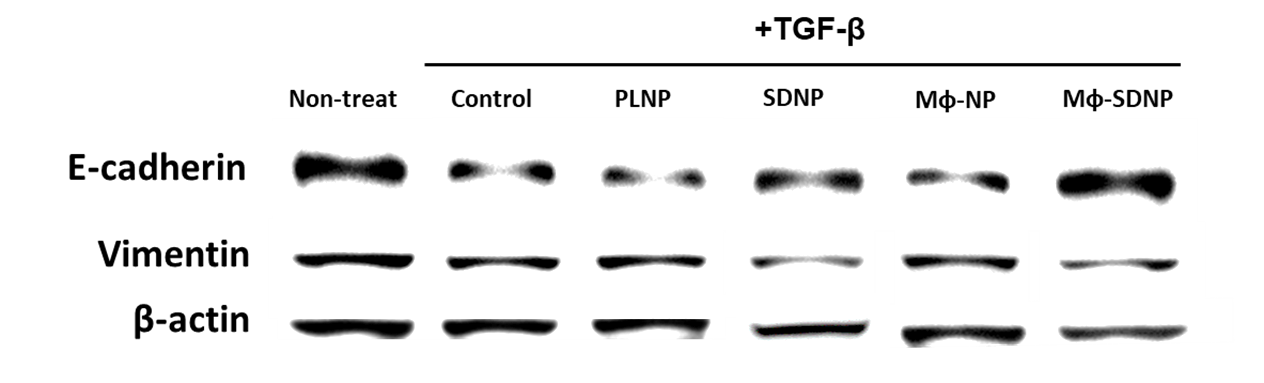
 Fig. S5.** Western blot images demonstrating inhibition of TGF-β-mediated epithelial-to-mesenchymal transition (EMT) with SD-208 loaded nanoparticles. Treatment with SDNP and Mϕ-SDNP to 4T1 cells recovered E-cadherin expression level, which was reduced by TGF-β. In addition, the expression level of vimentin, a mesenchymal cell marker, was decreased.


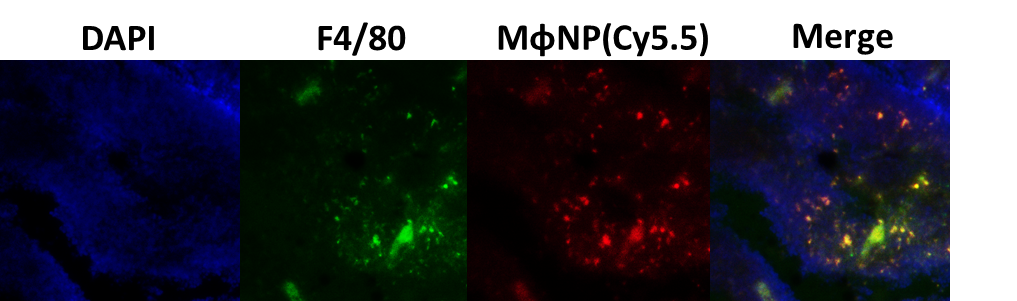
 **Fig. S6.** Immunostained tumor image demonstrating tumor-associated macrophage-targeting ability of MϕNP. Green signals represent tumor-associated macrophages (F4/80+), red signals represent Cy5.5-loaded MϕNP. It was shown that the MϕNPs penetrating inside the tumor tissue were well delivered to macrophages inside the tumor microenvironment.

**
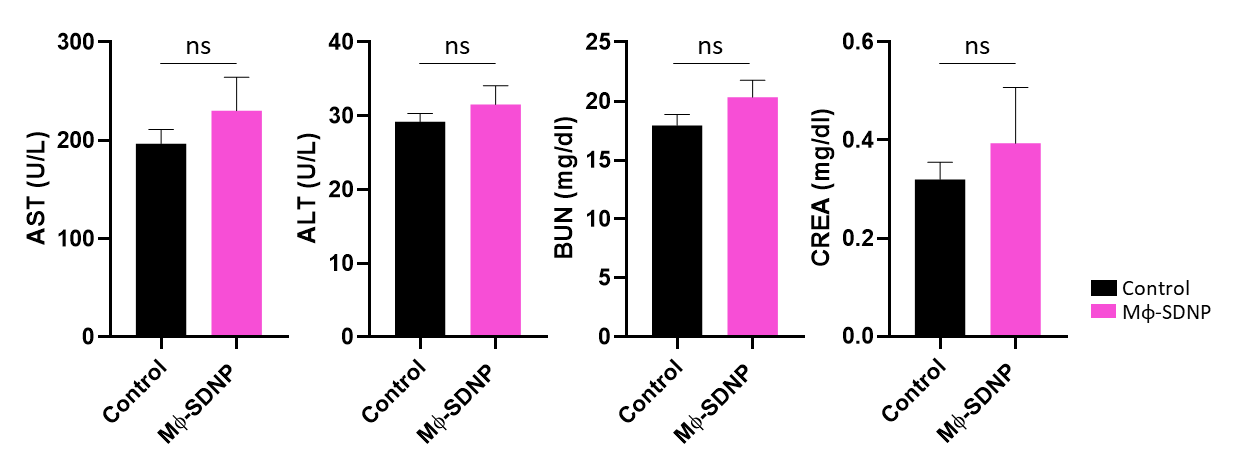
**

**Fig. S7.** In vivo toxicity evaluation of Mϕ-SDNP. Toxicity was analyzed by measuring the levels of enzymes reflecting the functions of the liver and kidneys, such as aspartate aminotransferase (AST), alanine aminotransferase (ALT), blood urea nitrogen (BUN), and creatinine (CREA) in the plasma of 4T1 tumor-bearing mice on the day of last injection. Data represent mean ± SD. ns = not significant difference. Statical analysis was followed by student’s T test (n =3).

**
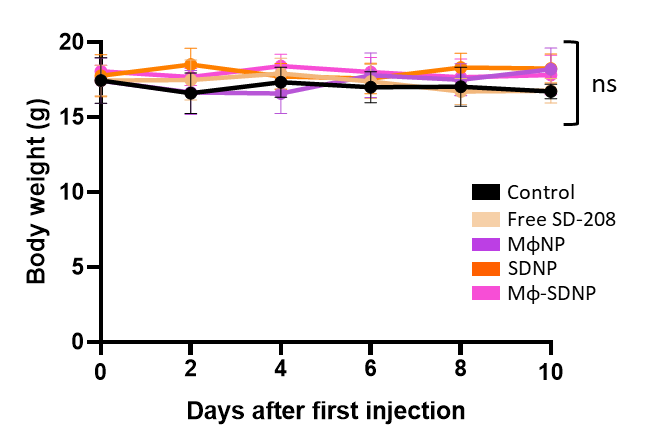
**

**Fig. S8.** Body weight profiles (n=5). Data represent mean ± SD. ns = not significant difference. Statical analysis was followed by two-way ANOVA with Boneferroni post-tests (n=5).


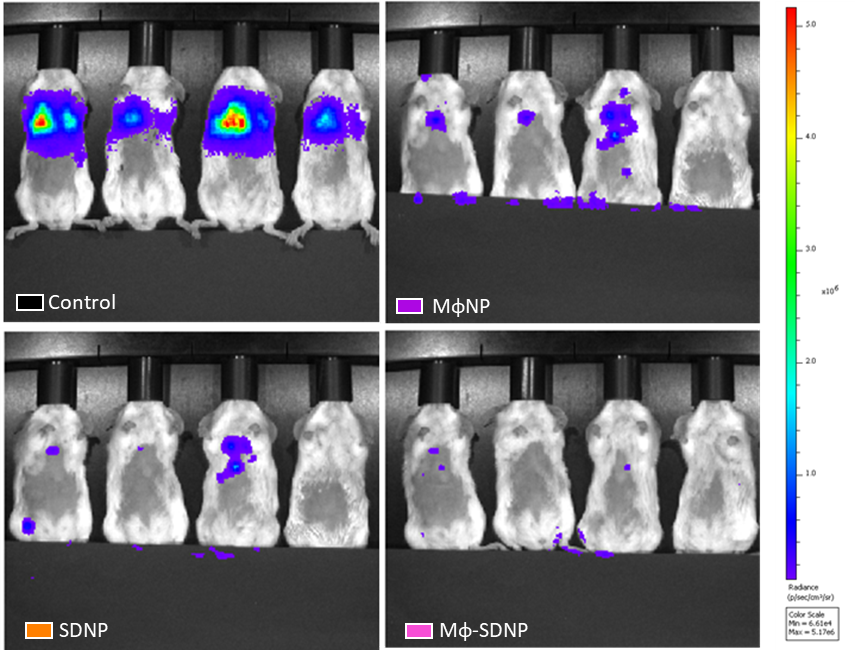


**Fig. S9.** In vivo luciferase imaging for verifying anti-metastasis efficacy. The luminescence signal represents the luciferase signal from tail-vein-injected 4T1-luc2 cells. Luciferin was injected with 150mg/kg concentration. Intravenously injected 4T1-luc2 cells showed accumulation in the lungs 10 days after inoculation, and each nanoparticle-administered group showed a decreased accumulation of cells in the lungs.


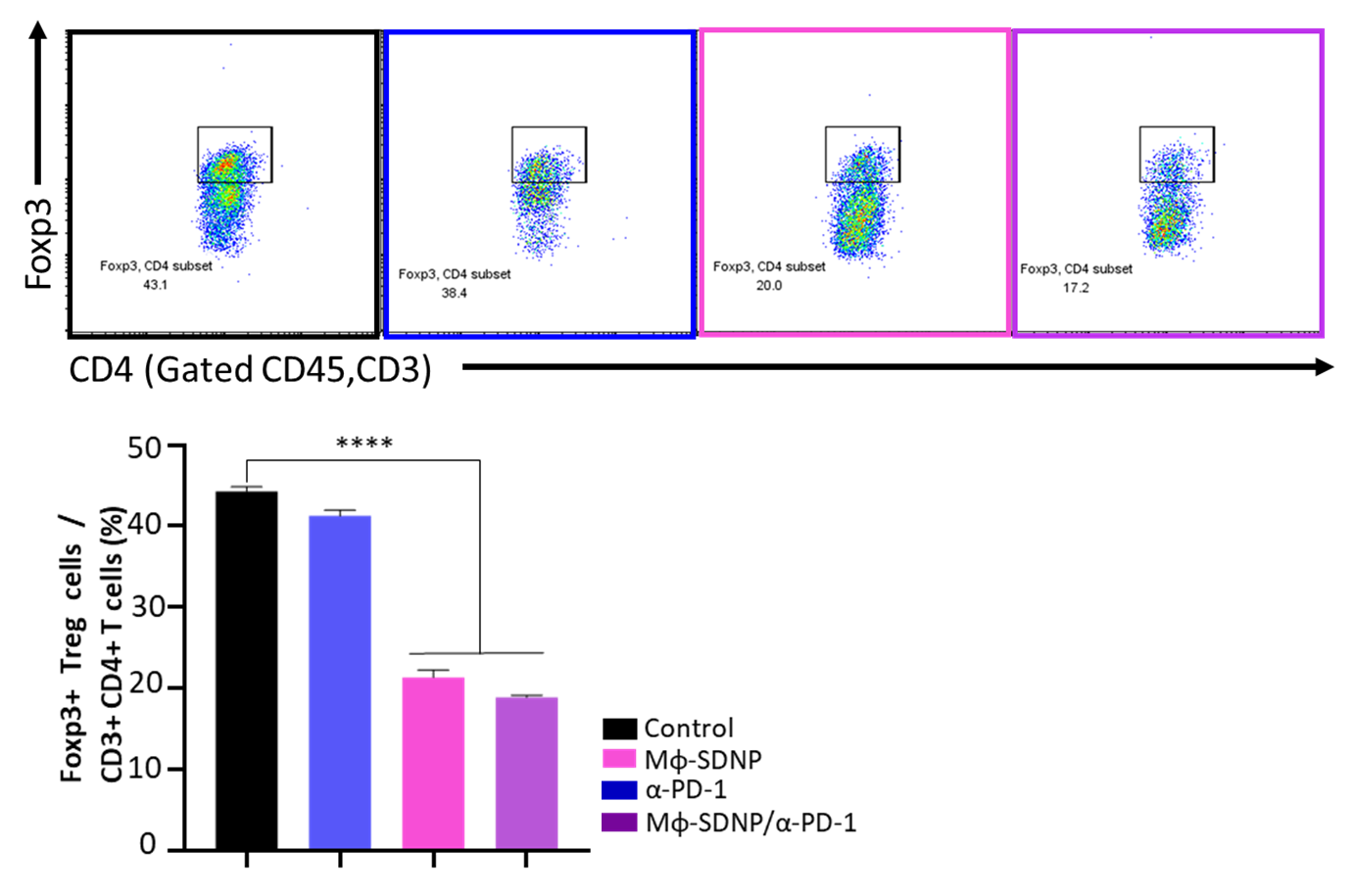


**Fig. S10.** Flow cytometric analysis of CD4 + Foxp3+ regulatory T cell population. The distribution of regulatory T cells within primary tumor tissue showed the lowest pattern in the combination treatment group with anti-PD-1 antibodies. Statical analysis was calculated by student’s t-test. ****p <0.001


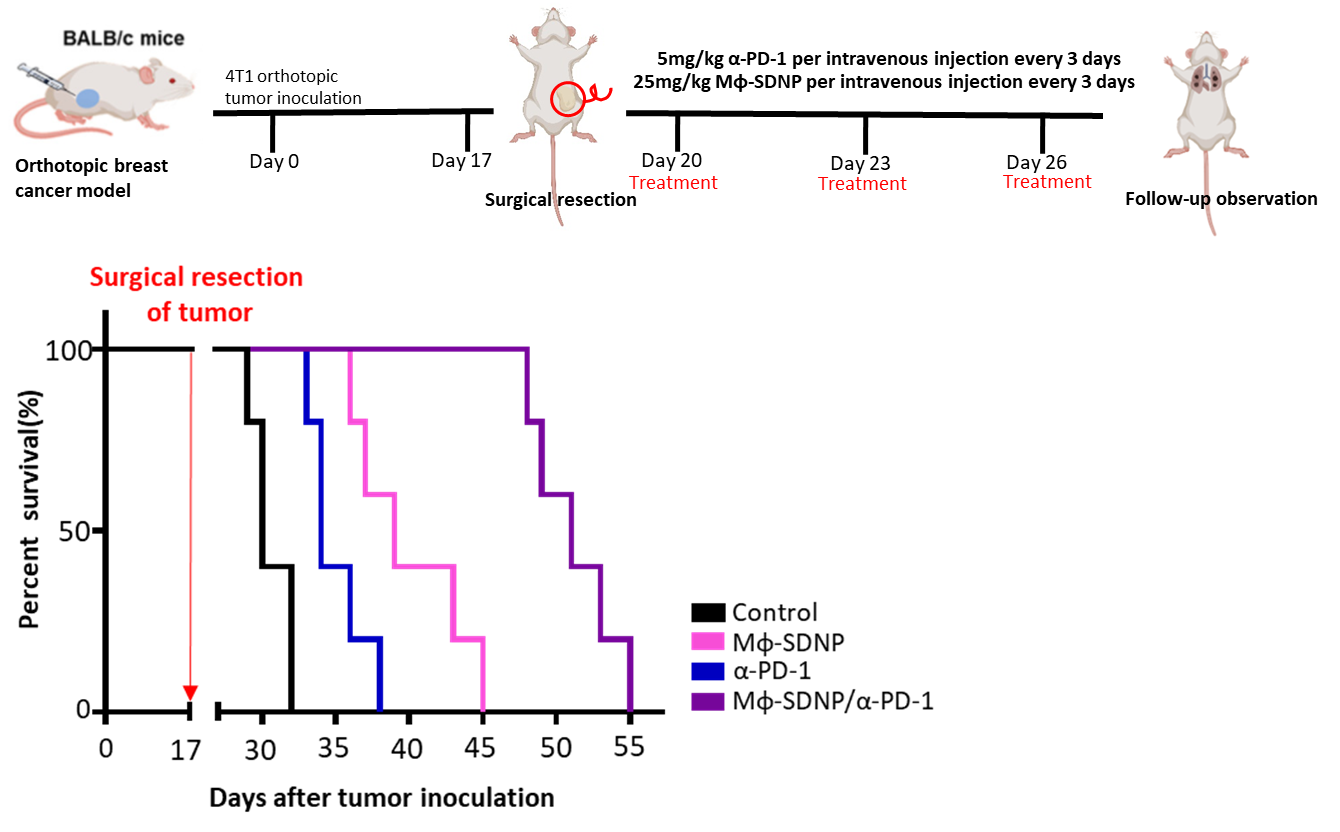


**Fig. S11.** In vivo anti-metastatic survival rate profile in combination therapy with anti-PD-1 antibody. After the first surgical resection of tumor tissue on day 17, it was confirmed that the response rate of the immune checkpoint inhibitor improved when nanoparticles and immune checkpoint inhibitors were administered together, resulting in a synergy effect.
